# Patient-Reported Outcomes of Functional Orthodontic Treatment for Skeletal Class II and III Malocclusions in Growing Patients: A Systematic Review

**DOI:** 10.7759/cureus.106573

**Published:** 2026-04-07

**Authors:** Dima M. Almrayati, Mohammad Y. Hajeer, Mowaffak A. Ajaj, Omar Hamadah, Jehad M. Kara-Boulad, Youssef Latifeh

**Affiliations:** 1 Department of Orthodontics, Faculty of Dentistry, University of Damascus, Damascus, SYR; 2 Department of Oral Medicine, Faculty of Dentistry, University of Damascus, Damascus, SYR; 3 Department of Orthodontics, Faculty of Dentistry, Mari Private University, Idlib, SYR; 4 Department of Psychiatry, Faculty of Medicine, University of Damascus, Damascus, SYR

**Keywords:** discomfort, functional appliances, functional impairment, oral health-related quality of life, pain, patient-reported outcome measures, skeletal class iii malocclusion, skeletal class ii malocclusion, speech difficulty, swallowing difficulties

## Abstract

Functional orthopedic appliances are widely used to manage skeletal Class II and Class III malocclusions in growing patients due to their well-established dentoskeletal effects. However, their impact on patient-reported outcome measures (PROMs) remains insufficiently investigated. PROMs, including pain, functional impairment, swallowing difficulties, and oral health-related quality of life (OHRQoL), are essential components of patient-centered care and are increasingly emphasized in contemporary orthodontic research. This systematic review aimed to critically evaluate the available evidence regarding PROMs associated with functional appliance therapy in growing individuals. A comprehensive electronic search was conducted in June 2025 across eight major databases without language restrictions. This was supplemented by manual screening of reference lists to ensure the comprehensive identification of studies. Eligible studies included randomized controlled trials and controlled clinical studies assessing PROMs in growing patients treated with fixed or removable functional appliances. The outcomes of interest included pain, functional limitations, swallowing difficulties, and OHRQoL, assessed using validated instruments such as Oral Health Impact Profile (OHIP-14), Child Perceptions Questionnaire (CPQ 11-14), and Child Oral Health Impact Profile (COHIP-19), as well as structured questionnaires. Risk of bias was evaluated using Cochrane Collaboration tools, and the certainty of evidence was assessed using the Grading of Recommendations Assessment, Development, and Evaluation (GRADE) approach. Eight studies met the inclusion criteria: seven randomized controlled trials (RCTs) and one prospective cohort study, totaling 447 participants. The findings suggest that functional appliance therapy is generally associated with mild-to-moderate pain, primarily during the initial adaptation phase, which resolves over time. Short-term functional disturbances, such as difficulties in mastication and speech, were also commonly reported but tended to diminish as patients adapted to the appliance. Swallowing difficulties were temporary and improved with continued use. The overall impact on OHRQoL was minimal, with no clinically significant deterioration reported across the included studies. However, the certainty of evidence ranged from low to very low, mainly due to methodological limitations, small sample sizes, heterogeneity in study designs, variability in appliance types, differences in outcome measures, and imprecision in results. Considerable clinical and methodological heterogeneity precluded a meta-analysis. In conclusion, functional appliance therapy for skeletal Class II and Class III malocclusions in growing patients appears to be well tolerated, with predominantly mild, transient, and self-limiting PROMs and no significant adverse impact on OHRQoL. Nevertheless, the limited certainty of evidence underscores the need for high-quality, adequately powered multicenter RCTs using standardized PROMs and longer follow-up periods. This systematic review was registered in the International Prospective Register of Systematic Reviews (PROSPERO) database during the early stages of its conduct (registration number: CRD420251166637).

## Introduction and background

Skeletal Class II malocclusion is one of the most common conditions in orthodontic clinics, especially among growing children and adolescents, and is characterized by increased overjet and a retruded mandible [1]. In contrast, Class III malocclusion is a rare condition but presents significantly greater challenges for orthodontists. Therefore, it is one of the cases that requires more careful analysis and an individualized treatment plan, particularly in growing patients [2].

Class II malocclusion is associated with an increased risk of incisor injuries and a higher likelihood of teasing and bullying, which can negatively affect the child's psychological well-being. Additionally, this malocclusion has been linked to adverse effects on oral health-related quality of life (OHRQoL) [3,4]. On the other hand, Class III malocclusion is associated with functional impairments, such as difficulties in chewing and speech. And it may also result in negative social experiences, including teasing and reduced self-esteem. These factors have similarly been shown to adversely affect OHRQoL [4,5].

Functional treatment is very important for the early management of both Class II and Class III skeletal malocclusions. In Class II malocclusion, functional appliances are designed to stimulate mandibular growth and improve sagittal discrepancies by posturing the lower jaw forward [6]. On the other hand, functional treatment of Class III malocclusion typically includes functional appliances that promote forward growth of the maxilla and restrict mandibular growth [7]. The optimal timing for functional treatment is typically during the pubertal growth spurt for Class II malocclusion and as early as possible during the mixed dentition phase for Class III malocclusion to maximize skeletal correction and treatment effectiveness.

While the objectives of functional treatment differ between Class II and Class III cases, the overall goal remains consistent: to utilize the patient's growth to correct skeletal discrepancies, enhance facial aesthetics, and improve oral function and quality of life. The effectiveness of these treatments depends on multiple factors, including the patient's age, severity of the malocclusion, appliance design, and patient compliance [8,9].

In recent years, the evaluation of orthodontic treatment outcomes has expanded beyond traditional clinical and cephalometric measures to include patient-reported outcome measures (PROMs), particularly those related to quality of life [10,11]. According to the WHO, quality of life means being fully well in body, mind, and social life, rather than simply being free from disease. [12]. According to Cunningham, OHRQoL refers to the impact of oral health conditions on an individual’s daily functioning, comfort, and psychosocial status [13]. Studies have shown conflicting results regarding the impact of functional orthodontic appliances on patients' quality of life. Čirgić et al. found that the Andersen activator was associated with considerable pain and discomfort during the early stages of treatment [14]. Conversely, Idris et al. reported that patients experienced only mild pain with the same appliance, which notably diminished over time [15]. In terms of psychological impact, Majanni et al. found that over half of the participants experienced moderate to severe reductions in self-confidence while using removable mandibular retractor (RMR) appliances till the end of treatment [16]. However, Saleh et al. have documented a significant and progressive improvement in patients’ confidence in public settings as treatment advanced [17].

To date, no systematic review has specifically examined the impact of functional treatment on PROMs, including OHRQoL, in patients with either Class II or Class III malocclusions. Therefore, the aim of this systematic review is to critically evaluate the available evidence regarding the impact of functional treatment for skeletal Class II and III malocclusions on PROMs.

## Review

Materials and methods

Eligibility Criteria

The review question and criteria for selecting studies were defined using the Population, Intervention, Comparison, Outcomes, and Study design (PICOS) framework. The population included healthy, growing male and female patients of any racial background presenting with skeletal Class II or Class III malocclusion and were treated using either fixed or removable functional orthopedic appliances. The intervention considered any functional therapy utilizing fixed or removable functional appliances. For comparison, studies were eligible if they involved either untreated patients or individuals who received a different type of functional appliance or orthopedic mechanics than the intervention group. In terms of study design, we included randomized controlled trials (RCTs) and comparative observational studies published up to October 2025. The outcomes evaluated were PROMs, including satisfaction, functional impairment, pain, discomfort, and OHRQoL, assessed using validated instruments such as the Visual Analogue Scale (VAS), Numerical Rating Scale (NRS), verbal rating scales, or ordinal Likert scales. Studies were excluded if they were case reports, case series, retrospective studies, in vitro experiments, animal studies, editorials, opinion articles, or technical notes focused solely on treatment procedures.

Search Strategy

A comprehensive electronic search was conducted across several major databases to identify studies published up to October 2025. The databases included PubMed, Web of Science, Scopus, Excerpta Medica database (Embase), Google Scholar, and the Cochrane Central Register of Controlled Trials (CENTRAL). In addition, clinical trial registries such as the WHO’s International Clinical Trials Registry Platform (ICTRP) and ClinicalTrials.gov were explored to capture trials that were completed, ongoing, or yet to be published. No restrictions were applied regarding gender, ethnicity, or language. The keywords used in the current review are given in Table 1, and the complete electronic search strategy is detailed in Appendix A.

**Table 1:** Keywords used in the electronic search

Components of the search strategy	Keywords and the related terms
Type of malocclusion	Skeletal class II, distal occlusion, mandibular retrognathia, class II, class III, Skeletal class III, mandibular prognathism, mesial occlusion.
Treatment planning	Functional treatment, growth modification, orthopedic treatment, mandibular advancement, mandibular enlargement.
Outcomes	Pain, Discomfort, Acceptance, self-confidence, Quality of life, happiness, Articulation, Bleeding, Chewing, Depression, Erosions, Functional impairment, Irritation, Irritating mucosa, Limitation, Mastication, OHRQoL, Oral ulcers, Oral Health related Quality of Life, Patient experiences, Patient Perception, patient-reported outcome measures, PROMs, Pressure, Restriction, Sleep, Soreness, Speech, Swelling, Tension, Self-esteem, edema, paresthesia.
Intervention	Removable functional appliances, Activator, Frankel regulator, Twin-block, Trainer, Bionator, Bite jumping appliance, Hotz, Dynamax, Bimler's appliance, Bass appliance, fixed functional appliances, AdvanSync, Forsus Fatigue-Resistant Device, Herbst, Jasper jumper.

Study Selection

Two independent reviewers (DMA and MYH) screened the studies to determine their eligibility for inclusion in this systematic review. Whenever disagreements arose, a third reviewer (MAA) was consulted. The initial screening focused on titles and abstracts, and studies that appeared potentially relevant or had insufficient information in their titles or abstracts were retrieved in full for detailed evaluation. Data extraction was carried out collaboratively by the two reviewers (DMA and MYH), with the third reviewer acting as an arbitrator in the event of any discrepancies. The information collected included general study information (authors, year of publication, and study location), methodological details (treatment comparisons), participant characteristics (sample size, age, and gender), orthodontic parameters (malocclusion classification), and study outcomes (type of scale used and time of evaluation). These details were compiled in summary tables for analysis. To reduce potential bias, any studies in which one of the review authors was also a co-author were independently assessed by two other reviewers who were not involved in the original study. This procedure was applied during both the data extraction and risk-of-bias assessment stages to ensure objectivity and transparency.

Assessment of the Risk of Bias in the Included Studies and Quality of Evidence

Two independent reviewers (DMA, MYH) evaluated the quality of the included studies using the Cochrane Risk of Bias 2.0 (RoB-2) tool for RCTs [18]. Each domain was classified as low, high, or some concerns. Any disagreements between the reviewers were resolved through discussion with a third reviewer (MAA). Non-randomized studies were assessed using the Risk of Bias in Non-randomized Studies of Interventions (ROBINS-I) tool, following Cochrane guidelines [19]. The Grading of Recommendations Assessment, Development, and Evaluation (GRADE) approach was used to assess the reliability of the conclusions and the strength of the evidence for outcomes [20]; the level of evidence was rated as high, moderate, low, or very low.

Results

Study Selection and Inclusion Into the Review

**Figure 1:**
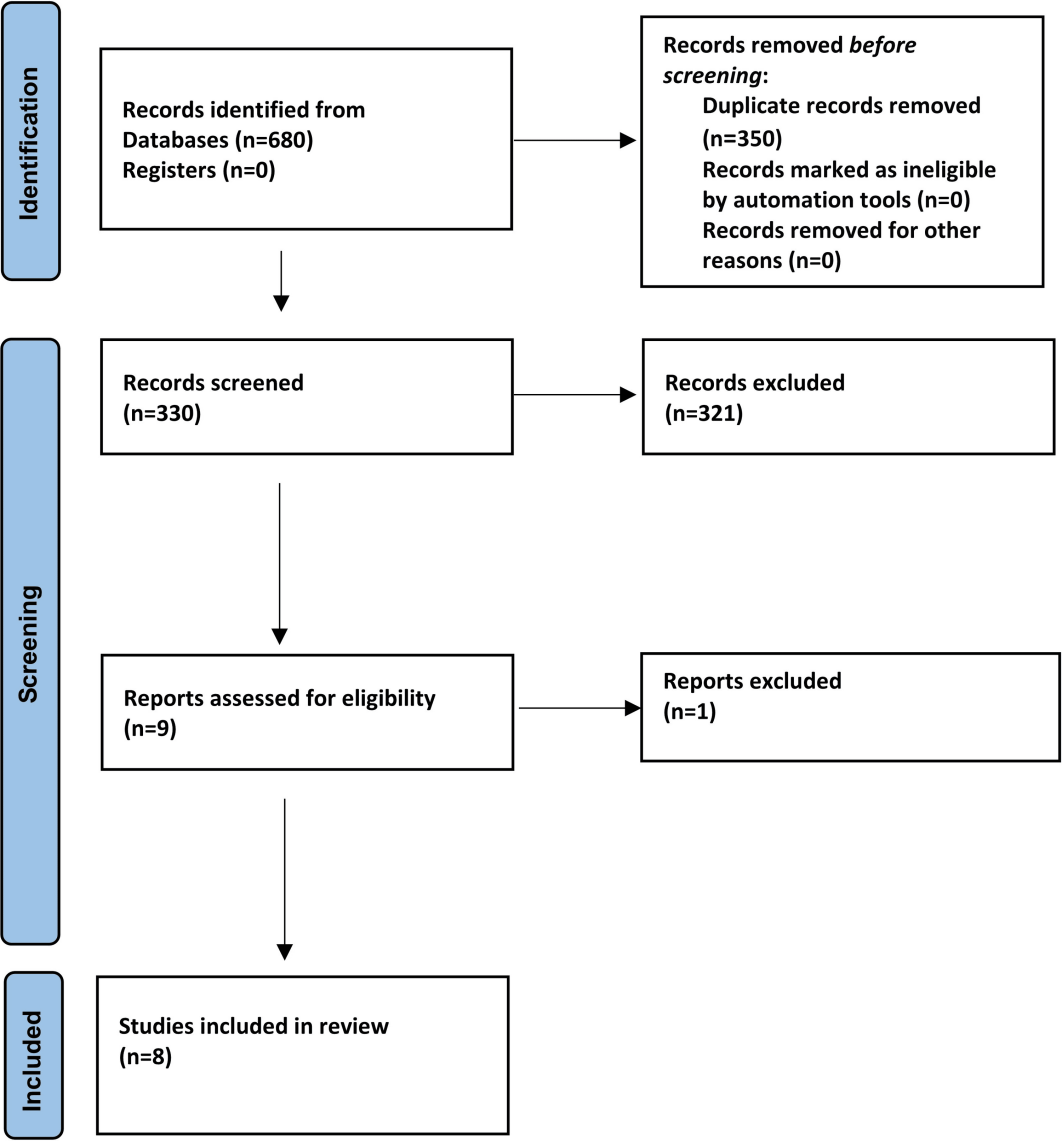
Preferred Reporting Items for Systematic Reviews and Meta-Analyses (PRISMA) flow diagram of the included studies.

The Preferred Reporting Items for Systematic Reviews and Meta-Analyses (PRISMA) flow diagram illustrating the screening, selection, and inclusion process of studies in this review is presented in Figure 1. A total of 680 records were identified through electronic database searches. After removing duplicates, 330 titles and abstracts were screened. Subsequently, the full texts of nine potentially relevant articles were assessed for eligibility. One study was excluded at the full-text review stage because it did not report numerical data suitable for inclusion in the qualitative synthesis; attempts to contact the authors for additional information were unsuccessful [14]. As a result, eight studies were included in this systematic review.

Characteristics of the Included Studies

Table 2 summarizes the characteristics of the eight studies included in this systematic review, comprising seven RCTs and one cohort study. A total of 447 participants, both males and females, were enrolled. Participant ages varied by malocclusion type: those with Class II malocclusion ranged from 10.6 to 16 years, while those with Class III malocclusion ranged from 7.5 to 15 years. Five of the eight studies focused on Class II malocclusion and examined the effects of functional orthodontic treatment on patient-reported outcomes [15,21-24]. The remaining three studies addressed Class III malocclusion [16,17,25].

**Table 2:** Characteristics of the included studies RCT: Randomized clinical trial; Act: Activator appliance; T4K: Trainer; RMR: Removable Mandibular Retractor; FR2: Frankel 2; TB: Twin-block appliance; ATB: Aesthetic Twin-block; BAIMT: Bone-Anchored Intermaxillary Traction; M: male; F: female; NR: Not reported; SME: slow maxillary expansion; RME: rapid maxillary expansion; CPQ-14: Child Perceptions Questionnaire; COHIP-19: Child Oral Health Impact Profile; OHIP-14: Oral Health Impact Profile

Study/setting	Study design	Type of appliances used	Number of patients (M/F); Age in years	Inclusion criteria	Time of evaluation	Outcomes
Class II malocclusion						
Idris et al., 2012 [15] Syria	RCT	Act vs. T4k	54 (28/26); -10.6 years	Skeletal Class II relationship (ANB > 4 degrees), Overjet ≥ 4 mm mandibular retrognathia	(T1) 7 days (T2) 14 days (T3) 3 months (T4) 6 months	Pain and functional impairments, using standardised questionnaires.
Campbell et al., 2020 [23] United Kingdom	RCT	TB vs. FR2	60 (27/33); 11-14 year	Overjet ≥ 8mm	(T1) before treatment (T2) at the end of active treatment	Child OHRQoL (CPQ11-14)
Pacha et al., 2023 [22] United Kingdom	RCT	TB vs. Hanks Herbst	80 (40/40); 10-14 years	Class II Division 1 incisor relationship overjet ≥7 mm	(T1) before treatment (T2) at the end of active treatment	COHIP-19
Pakkhesal et al., 2023 [21] Iran	Cohort	TB vs.FR2	88 (36/52); 10- 16 years	Skeletal Class II pattern (ANB > 4) mandibular retrognathia	(T1) before treatment. (T2) 1 week. (T3) 1 month. (T4) 3 months. (T5) 6 months.	OHIP-14 score
Alsilq et al., 2025 [24]	RCT	ATB vs. CTB	52(19/33); 12.33 ±0.77	Skeletal Class II relationship (ANB > 4 degrees) mandibular retrognathia	(T1) 7 days (T2) 14 days (T3) 3 months (T4) 6 months	Pain and functional impairments, using standardised questionnaires.
Class III malocclusion						
Saleh et al., 2013 [17] Syria	RCT	RMR vs. control non-treated group	33 (17/16); 7.5±1.33 years	Skeletal Class III relationship	(T1) 7 days (T2) 14 days (T3) 6 weeks (T4) 3 months (T5) 6 months	Pain and functional impairments, using standardised questionnaires.
Majanni et al., 2020 [16] Syria	RCT	RMR vs. BAIMT	56 (29/27); 11.46±0.89 years	Skeletal Class III relationship (-4	(T1) 1 day (T2) 7 days (T3) 6 weeks (T4) 3 months (T5) 6 months	Functional impairments, pain, and using standardised questionnaires
Zakaria et al., 2025 [25] Syria	RCT	RMR+SME vs. BAIMT+RME	24(13/11) 12-15 years	Skeletal Class III relationship (-4	(T1) 1 day (T2) 7 days (T3) 4 weeks (T4) 3 months (T5) 6 months	Functional impairments, pain, and using standardised questionnaires

Among the Class II studies, four investigated the twin-block appliance, comparing it with the Frankel II [21,23], the Hank Herbst appliance [22], and the aesthetic twin-block [24]. The Activator appliance was analyzed in a study that compared it with the trainer [15]. Regarding Class III malocclusion, the studies examined the use of an RMR appliance, comparing its effectiveness with that of bone-anchored intermaxillary traction systems [16,25] and the natural growth progression observed in untreated patients [17].

Regarding outcome measures, five studies evaluated pain and functional impairment associated with the functional appliance using standardized, self-reported questionnaires [15-17,24,25]. Additionally, one study utilized the Oral Health Impact Profile (OHIP-14) to assess the broader effect of treatment OHRQoL [21]. The remaining two studies also evaluated OHRQoL, using the Child Oral Health Impact Profile (COHIP-19) [22] and the Child Perceptions Questionnaire (CPQ11-14) [23].

Risk of Bias in the Included Studies

**Figure 2:**
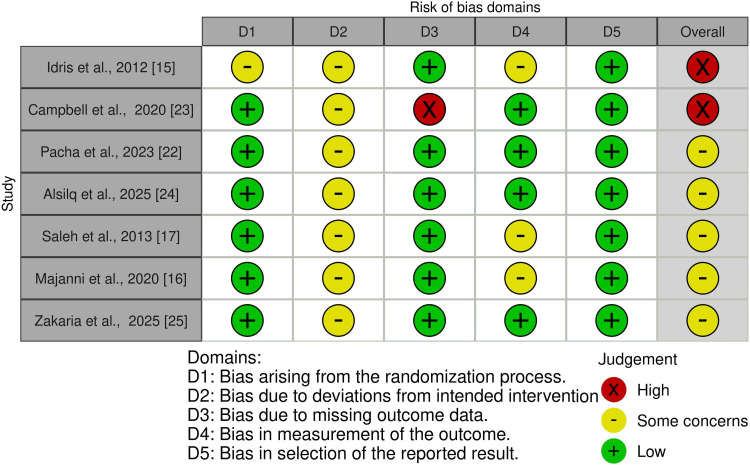
Risk of bias summary of randomized clinical trials

**Figure 3:**
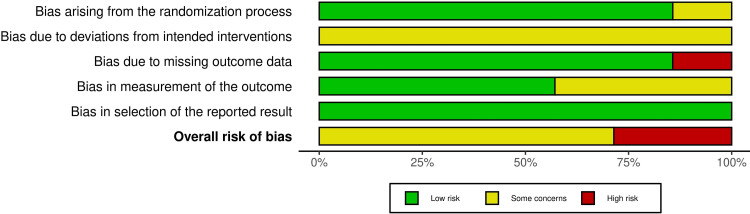
The overall risk of bias score for each field of randomized clinical trials

**Figure 4:**
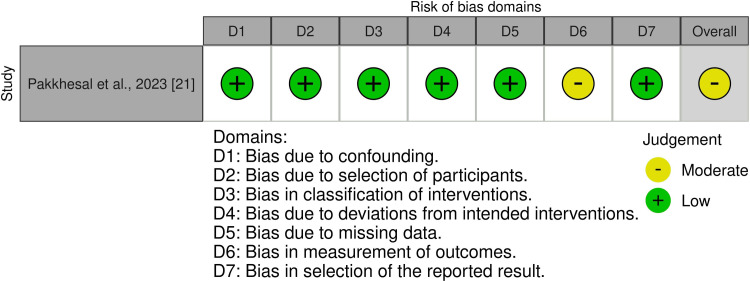
Risk of bias summary of the cohort study

The risk of bias for the seven included RCTs is presented in Figure 2, and an overall summary across all bias domains is shown in Figure 3. Detailed evaluations and justifications are available in Appendix B. Of the seven RCTs, two were rated as having a "high risk of bias" [15,23], while the remaining five were categorized as having "some concerns" [16,17,22,24,25]. In contrast, Figure 4 depicts the risk-of-bias assessment for the included cohort study. The cohort was judged to have a "moderate risk of bias" [21]. Additional information and rationale for these assessments are provided in Appendix C.

Effect of the Intervention

As a meta-analysis could not be performed due to considerable heterogeneity in outcome measures, follow-up durations, and age ranges, the findings of the included studies were synthesized narratively, emphasizing observed trends in pain, swallowing, and speech impairment, as well as oral health-related quality of life.

Pain Associated with Class II Functional Appliances

Two studies have specifically examined pain during functional orthodontic treatment for Class II malocclusion. Idris et al. assessed pain levels in patients treated with either the activator or the trainer appliance, using a four-point Likert scale (1 = not at all, 2 = little, 3 = much, 4 = very much). The results demonstrated a consistent decrease in pain intensity over time in both groups (Table 3). Pain was reported as little across all evaluation time points: seven days following appliance insertion (T1), two weeks (T2), three months (T3), and six months (T4). Mean scores declining from T1 to T4 for the activator group (x̄ = 1.68, x̄ = 1.57, x̄ = 1.18, and x̄ = 1.07, respectively). And the trainer group (x̄ = 1.42, x̄ = 1.46, x̄ = 1.15, and x̄ = 1.15, respectively). Although the Activator group consistently reported slightly higher pain scores than the Trainer group, the differences were not statistically significant (P = 0.279, 0.516, 0.793, and 0.609, respectively) [15]. Similarly, Alsilq et al. evaluated pain experienced during treatment with the twin-block and the aesthetic twin-block appliances using the same four-point Likert scale across four time points (T1-T4). Their findings demonstrated a steady and clinically meaningful decline in pain intensity for both appliances, with mean scores decreasing progressively from T1 to T4 (T1: x̄ = 2.88, x̄ = 2.18, T2: x̄ = 2.0, x̄ = 1.58, T3: x̄ = 0.98, x̄ = 0.66, and T4: x̄ = 0.6, x̄ = 0.51, respectively). Despite this gradual improvement, no statistically significant differences were observed between the two appliance designs throughout the evaluation period [24]. These findings are based on low-certainty evidence according to the GRADE approach (Table 4).

**Table 3:** Main findings of the included studies ACT: Activator appliance; TB: Twin-block appliance; ATB: Aesthetic Twin-block ; T4K®: Trainer; FR: Frankel; HH: Hanks Herbst; RMR: Removable mandibular retractor; CPQ-14: Child Perceptions Questionnaire; COHIP-19: Child Oral Health Impact Profile; OHIP-14: Oral Health Impact Profile; NA: Not available

Authors and Year	Appliance	Pain	Swallowing Impairment	Speech Impairment	OHRQoL CPQ11-14/ COHIP-19/ OHIP-14
Idris et al., 2012 [15]	ACT	1 week: x̄= 1.68, 2 weeks: x̄= 1.57, 3 months: x̄= 1.18, 6 months: x̄= 1.07	1 week: x̄= 1.43, 2 weeks: x̄= 1.39, 3 months: x̄= 1.04, 6 months: x̄= 1.00	1 week: x̄=1.96, 2 weeks: x̄=1.79, 3 months: x̄=1.36, 6 months: x̄=1.18	NA
	T4K®	1 week: x̄= 1.42, 2 weeks: x̄= 1.46, 3 months: x̄= 1.15, 6 months: x̄= 1.15	1 week: x̄= 1.23, 2 weeks: x̄= 1.23, 3 months:x̄= 1.08, 6 months: x̄= 1.04	1 week: x̄=3.58, 2 weeks: x̄=3.27, 3 months: x̄=3.23, 6 months: x̄=3.00	NA
Campbell et al., 2020 [23]	FR	NA	NA	NA	x̄=46.0
	TB	NA	NA	NA	X̄= 45.4
Pacha et al., 2023 [22]	TB	NA	NA	NA	X̄= 24.7 ± 13
	HH	NA	NA	NA	X̄= 24.7 ± 11.7
Pakkhsal et al., 2023 [21]	TB	NA	NA	NA	1 week: x̄= 15.97 ± 6.15, 1 month: x̄=15.59 ± 6.18, 3 months: x̄=12.43 ± 5.85, 6 months: x̄= 10.36 ± 4.84
	FR	NA	NA	NA	1 week: x̄=10.13 ± 4.69, 1 month: x̄=8.18 ± 3.96, 3 months: x̄=8.18 ±3.96, 6 months: x̄= 5.04 ± 2.37
Alsilq et al., 2025 [24]	TB	1 week: x̄= 2.88, 2 weeks: x̄= 2.0, 3 months: x̄= 0.98, 6 months: x̄= 0.6	NA	1 week: x̄= 3.3, 2 weeks: x̄= 3.08, 3 months: x̄= 2.58, 6 months: x̄= 2.3	NA
	ATB	1 week: x̄= 2.18, 2 weeks: x̄= 1.58, 3 months: x̄= 0.66, 6 months: x̄= 0.51	NA	1 week: x̄= 2.88, 2 weeks: x̄= 2.26, 3 months: x̄= 1.76, 6 months: x̄= 1.42	NA
Saleh et al., 2013 [17]	RMR	1 week: x̄= 1.22, 2 weeks: x̄= 1.13, 6 weeks: x̄= 1.06, 3 months: x̄= 1.03, 6 months: x̄= 1.03	1 week: x̄= 1.81, 2 weeks: x̄= 1.31, 6 weeks: x̄= 1.28, 3 months: x̄= 1.09, 6 months: x̄= 1.09	1 week: x̄=1.88, 2 weeks: x̄=1.47, 6 weeks: x̄=1.38, 3 months: x̄=1.25, 6 months: x̄=1.09	NA
Majanni et al., 2020 [16]	RMR	1 day: x̄= 0.58 ± 0.9, 1 week: x̄= 0.47 ± 0.84, 6 weeks: x̄= 0.37 ± 0.68, 3 months: x̄= 0.21 ± 0.42, 6 months: x̄= 0.11 ± 0.32	1 day: x̄= 0.58, 1 week: x̄= 0.47, 6 weeks: x̄= 0.37, 3 months: x̄= 0.21, 6 months: x̄= 0.11	NA	NA
Zakaria et al., 2025 [25]	RMR + SME	1 day: 50% none, 33.3% mild, 8.3% medium, 8.3% severe; 1 week: 75% none, 16.75 mild, 8.3% severe; 4 weeks: 66.7% none, 8.3% mild, 25% medium; 3 months: 50% none, 25% mild, 16.7% medium, 8.3% severe; 6 months: 75% none, 25% mild	1 day: 16.7% none, 25% mild, 16.7% medium, 41.7% severe; 1 week: 50% none, 25% mild, 8.3% medium, 16.7% severe; 4 weeks: 75% none, 16.7% mild, 8.3% severe; 3 months: 91.7% none, 8.3% medium; 6 months: 100% none	1 day: 8.3% none, 83.3% medium. 8.3% severe; 1 week: 16.7% none, 16.7% mild, 58.3% medium, 8.3% severe; 4 weeks: 58.3% none, 8.3% mild, 33.3% medium; 3 months: 58.3% none, 25% mild, 8.3% medium, 8.3% severe; 6 months: 66.7% none, 25% mild, 8.3% medium	NA

**Table 4:** Summary of the findings from the included studies according to the Grading of Recommendations Assessment, Development and Evaluation (GRADE) approach. High quality: Further research is very unlikely to change our confidence in the effect estimate. Moderate quality: Further research is likely to have an important impact on our confidence in the effect estimate and may change it. Low quality: Further research is very likely to have an important impact on our confidence in the effect estimate and is likely to change it. Very low quality: We are very uncertain about the estimate RCT: Randomized clinical trial, OHRQoL: Oral Health–Related Quality of Life, CPQ (11-14): Child Perceptions Questionnaire, COHIP-19: Child Oral Health Impact Profile, OHIP-14: Oral Health Impact Profile, ACT: Activator, TB: Twin-block, ATB: Aesthetic twin-block; T4K®: Trainer. RMR: Removable mandibular retractor; FR2: Frankel 2, HH: Hanks Herbst. ^a^ Downgraded due to risk of bias; ^b ^Downgraded due to small sample size; ^c^ Downgraded due to moderate heterogeneity between studies.

Quality assessment criteria							Summary of findings				Comments
Outcomes	Study design	Risk of bias	Inconsistency	Indirectness	Imprecision	Other considerations	Number of patients (trials)	Effects		Certainty	
								Absolute (95% CI)	Relative (95% CI)		
Class II											
Pain											
Pain changes with functional treatment	RCT	Serious	Serious	Not serious	Not serious	None	106 (2)	-	-	Low ⊕⊕⊖⊖^a,c^	Evidence suggests that functional therapy with ACT, TB, ATB, and TK4 causes mild, gradually decreasing pain, although the evidence quality is low.
Swallowing impairment											
Swallowing impairment changes with functional treatment	RCT	Serious	Not serious	Not serious	Serious	None	54 (1)			Low ⊕⊕⊖⊖^a,b^	Evidence suggests that functional therapy with ACT and TK4 results in mild, gradually decreasing swallowing impairment, although the quality of the evidence is low.
Speech impairment											
Speech impairment changes with functional treatment	RCT	Serious	Serious	Not serious	Not serious	None	106 (2)	-	-	Low ⊕⊕⊖⊖^a,c^	Evidence suggests that functional therapy with ACT, TB, ATB, and TK4 results in mild, gradually decreasing speech impairment, although the quality of the evidence is low.
OHRQoL											
Change in CPQ 11-14 score after functional treatment	RCT	Serious	Not serious	Not serious	Serious	None	60 (1)	-	-	Low ⊕⊕⊖⊖^a,b^	Evidence suggests that functional therapy with TB and FR2 causes a mild to moderate impact on quality of life by the end of treatment, although the evidence quality is low.
Change in COHIP-19 score changes after functional treatment	RCT	Serious	Not serious	Not serious	Serious	None	80 (1)	-	-	Low ⊕⊕⊖⊖^a,b^	Evidence suggests that functional therapy with HH and FR2 causes a moderate impact on OHRQoL at the end of treatment, although the evidence quality is low.
Change in OHIP-14 score after functional treatment	Cohort	Serious	Not serious	Not serious	Serious	None	88 (1)	-	-	Very Low ⊕⊖⊖⊖^a,b^	Evidence suggests that functional therapy with FR2 causes a better impact on OHRQoL during treatment than TB, although the evidence quality is very low.
Class III											
Pain											
Pain changes with functional treatment	RCT	Serious	Serious	Not serious	Not serious	None	113 (3)	-	-	Low ⊕⊕⊖⊖^a,c^	Evidence suggests that functional therapy with RMR causes mild, gradually decreasing pain, although the evidence quality is low.
Swallowing impairment											
Swallowing impairment changes with functional treatment	RCT	Serious	Serious	Not serious	Not serious	None	113 (3)	-	-	Low ⊕⊕⊖⊖^a,c^	Evidence suggests that functional therapy with RMR causes a progressive decrease in swallowing impairment throughout treatment, although the quality of the evidence is low.
Speech impairment											
Speech impairment changes with functional treatment	RCT	Serious	Serious	Not serious	Not serious	None	57 (2)	-	-	Low ⊕⊕⊖⊖^a,c^	Evidence suggests that functional therapy with RMR causes a progressive decrease in speech impairment throughout treatment, although the quality of the evidence is low.

Pain Associated with Class III Functional Appliances

In a study conducted by Saleh et al., the pain associated with functional orthodontic treatment using the RMR was assessed using a four-point Likert scale at five time points: seven days following appliance insertion (T1), two weeks (T2), six weeks (T3), three months (T4), and six months (T5). The results demonstrated a progressive decrease in pain intensity throughout treatment, with mean scores declining from T1 to T5 (x̄ = 1.22, x̄ = 1.13, x̄ = 1.06, x̄ = 1.03, and x̄ = 1.03, respectively) [17]. Also, Majanni et al. measured pain during treatment with RMR, assessing it at multiple follow-up intervals: one day (T1), one week (T2), six weeks (T3), three months (T4), and six months after the initiation of treatment (T5). Pain was measured using a standardized, validated, four-point Likert scale. The results demonstrated a progressive decline in pain intensity over time, with complete disappearance reported by the six-month follow-up (x̄ = 0.58 ± 0.9, x̄ = 0.47 ± 0.84, x̄ = 0.37 ± 0.68, x̄ = 0.21 ± 0.42, x̄ = 0.11 ± 0.32, respectively) [16]. In the study by Zakaria et al. [25], pain associated with the RMR used in combination with slow maxillary expansion (SME) was generally mild and transient. It was measured using a four-point Likert scale. One day after appliance insertion (T1), 50% of patients reported no pain, 33.3% reported mild pain, 8.3% reported moderate pain, and one participant (8.3%) reported severe pain. After one week (T2), the proportion of pain-free patients increased to 75%, 16.7% reported mild pain, and severe pain remained uncommon (1/12, 8.3%). By one month (T3), most participants (66.7%) reported no pain, 25% reported medium pain, and 8.3% (1/12) reported mild pain. At three months, the pain remained limited, with 50% reporting no pain, 25% mild pain, 16.7% medium pain, and 8.3% (1/12) severe pain. By six months, the majority of patients (75%) were pain-free, while the remaining 25% reported only mild pain, indicating an overall trend toward decreasing discomfort over time. These findings are based on low-certainty evidence according to the GRADE approach.

Quality of Life Associated with Class II Functional Appliances

Three studies have investigated the impact of functional treatment with Class II appliances on patients’ OHRQoL. Campbell et al. conducted a comparative study between the Frankel II appliance and a modified Twin-block, using the CPQ 11-14; higher scores indicate worse OHRQoL. Their results showed that both appliances had a mild-to-moderate impact on quality of life by the end of treatment, and there was no statistically significant difference between the two groups (P = 0.78). The mean CPQ 11-14 scores were 46.0 for the Frankel group and 45.4 for the twin-block group [23].

Also, Pacha et al. evaluated the OHRQoL of children treated with twin-block and Herbst appliances using the COHIP-19 questionnaire at baseline and immediately post-treatment; higher scores indicate worse OHRQoL. Both groups experienced a moderate impact on OHRQoL at the end of treatment, with mean scores of 24.7 ± 13 and 24.7 ± 11.7, respectively, and no significant difference between the appliances (P = 0.9) [22].

Twin-block and Frankel II appliances, measured with OHIP-14 (higher scores indicate worse OHRQoL) at one week, one month, three months, and six months. Scores decreased over time in both groups, with the Frankel II consistently showing significantly better outcomes than the twin-block at all intervals (p < 0.001). Specifically, twin-block scores decreased from (x̄ = 15.97 ± 6.15 at one week to x̄ = 10.36 ± 4.84 at six months), while Frankel II scores decreased from (x̄ = 10.13 ± 4.69 at one week to x̄ = 5.04 ± 2.37 at six months) [21]. The strength of evidence for this finding ranged from low to very low according to the GRADE approach.

Swallowing and Speech Impairment with Class II Functional Appliances

According to Idris et al., both functional appliances caused a mild impairment in swallowing. Difficulty swallowing was assessed on a four-point Likert scale (higher scores indicate greater difficulty). However, the activator was associated with greater difficulty compared to the trainer during the initial evaluation periods: T1 (x̄ = 1.43, x̄ = 1.23, respectively) and T2 (x̄ = 1.39, x̄ = 1.23, respectively). This impairment significantly improved from the third time point onward, becoming comparable to the trainer, with no statistically significant difference between the two appliances (P=0.986, P=0.987, respectively). Regarding speech impairment, both appliances demonstrated a progressive reduction over time. Speech was assessed using the same four-point Likert scale. Patients treated with the activator reported lower mean difficulty scores compared to those using the trainer at each time point (T1: x̄ = 1.96, x̄ = 3.58; T2: x̄ = 1.79, x̄ = 3.27; T3: x̄ = 1.36, x̄ = 3.23; T4: x̄ = 1.18, x̄ = 3.00, respectively), with the differences reaching statistical significance at all evaluation time points (P = 0.000) [15]. Similarly, Alsilq et al. evaluated speech impairment experienced during treatment with the twin-block and the aesthetic twin-block appliances using the same four-point Likert scale. Their findings demonstrated a steady and clinically meaningful decline in impairment for both appliances, with mean scores decreasing progressively from T1 to T4 (T1: x̄ = 3.3, x̄ = 2.88, T2: x̄ = 3.08, x̄ = 2.26, T3: x̄ = 2.58, x̄ = 1.76, T4: x̄ = 2.3, x̄ = 1.42, respectively). Despite this gradual improvement, the twin-block caused greater speech impairment, with statistically significant differences observed from T2 to T4 (P = 0.013, P = 0.004, P = 0.001) [24]. These findings are based on low-certainty evidence according to the GRADE approach (Table 4).

Swallowing and Speech Impairment with Class III Functional Appliances

Saleh et al. reported that RMR caused some degree of swallowing difficulties during the first week of treatment. Swallowing difficulty was assessed using a four-point Likert scale. However, these difficulties gradually decreased over the evaluation periods from T1 to T5, with mean scores of (x̄ = 1.81, x̄ = 1.31, x̄ = 1.28, x̄ = 1.09, and x̄ = 1.09, respectively). However, the difficulties were not statistically significant at any time point [17]. Similarly, Majanni et al. observed mild swallowing difficulties during the initial week of treatment, which progressively declined throughout therapy. Swallowing difficulty was assessed using a four-point Likert scale. The mean scores decreased consistently across the evaluation periods, from (x = 1.05 ± 0.97) to (x = 0.95 ± 0.91, x = 0.68 ± 0.89, x = 0.47 ± 0.61, and x = 0.32 ± 0.48, respectively). And this impairment was not statistically significant [16].

Zakaria et al. [25] reported that the use of the RMR in combination with SME was initially associated with swallowing difficulties, which were evaluated using a four-point Likert scale. On the first day following appliance insertion (T1), only 16.7% of patients reported no difficulties, while 25% experienced mild, 16.7% medium, and 41.7% severe difficulties. By the end of the first week (T2), the proportion of patients without swallowing difficulties increased to 50%, with 25% reporting mild, 8.3% medium, and 16.7% severe difficulties. At one month (T3), 75% of participants reported no swallowing difficulties, 16.7% reported mild difficulties, and 8.3% reported severe difficulties. After three months (T4), the vast majority (91.7%) reported no difficulties, while 8.3% experienced medium difficulty. By six months (T5), all participants (100%) were free of any swallowing difficulties, indicating a progressive resolution of initial discomfort over time.

Regarding speech impairment, Saleh et al. reported a gradual decrease in speech impairment over time assessed using the same four-point Likert scale, with mean scores declining from T1 to T5 (x̄ = 1.88, x̄ = 1.47, x̄ = 1.38, x̄ = 1.25, and x̄ = 1.09, respectively); however, no statistically significant differences were observed between the five time points [17].

Zakaria et al. [25] found that wearing the RMR combined with SME was associated with early speech difficulties, evaluated using a four-point Likert scale. One day post-insertion (T1), most participants reported medium difficulty (83.3%), 8.3% reported severe difficulty, and 8.3% reported no difficulty. By one week (T2), speech difficulties had started to improve, with 16.7% reporting no difficulty, 16.7% mild difficulty, 58.3% medium difficulty, and 8.3% severe difficulty. At one month (T3), over half of the participants (58.3%) reported no speech difficulties, while 33.3% continued to report medium difficulty and 8.3% mild difficulty. Similar findings were observed at three months (T4), with 58.3% reporting no difficulty, 25% mild, 8.3% medium, and 8.3% severe. By six months (T5), speech difficulties had largely resolved: 66.7% of patients reported no difficulties, 25% mild difficulties, and 8.3% moderate difficulties, indicating gradual improvement over time. These findings are based on low-certainty evidence according to the GRADE approach.

Discussion

This review represents the first systematic synthesis of patient-reported outcomes for functional orthodontic appliances in skeletal Class II and Class III malocclusions. A total of eight clinical studies met the eligibility criteria, comprising seven randomized controlled trials and one cohort study, with 447 participants. Of these, five studies focused on Class II malocclusions, whereas three were conducted on Class III cases. The appliances assessed across the included studies varied considerably, including the twin-block, aesthetic twin-block, activator, Frankel II, Herbst, trainer appliance, and RMR. Patient-reported outcomes, including pain, functional impairment, and OHRQoL, were assessed using validated self-report measures (OHIP-14, COHIP-19, CPQ11-14) and customized questionnaires. Due to heterogeneity in outcome measures and follow-up periods, a quantitative meta-analysis was not feasible; therefore, the findings were synthesized narratively, providing a structured overview of current evidence and highlighting key methodological gaps for future research.

Risk of Bias Across Studies and Additional Analyses

Risk-of-bias assessments revealed methodological concerns that limit confidence in the effect estimates. Two randomized trials were judged to have an overall high risk of bias [15,23], while the remaining RCTs were rated as having some concerns [16,17,22,24,25], most commonly due to unblinded outcome assessment and incomplete reporting, as detailed in Appendix B. The single-cohort study was judged to have a moderate risk of bias [21], primarily because outcome measurement was not fully protected from knowledge of the intervention. And because outcome assessment was not fully blinded, the risk of detection bias remains. Therefore, the findings, supported by studies with varying levels of internal validity, should be viewed as indicative rather than conclusive.

Patient-Reported Outcomes Related to Functional Treatment

Across the reviewed studies, functional orthodontic appliances for both Class II and Class III cases were generally well tolerated. Most patients reported only mild, short-term discomfort or functional difficulties, and adaptation was a consistent pattern over time.

Pain associated with appliances such as the activator, twin-block, aesthetic twin-block, trainer, and RMR was consistently low, typically corresponding to the “a little” or "mild" category on the applied scales, and decreased progressively during follow-up. This improvement is plausibly explained by gradual neuromuscular and soft tissue adaptation to the altered mandibular position [15-17,24,25].

In terms of OHRQoL, the overall effect of functional appliances appeared limited. For Class II appliances, most studies found no statistically or clinically significant differences among the commonly used appliances, including the twin block, Herbst, and Frankel II, with effects generally ranging from mild to moderate [22,23]. A notable exception was reported by Pakkhesal et al., who observed consistently higher OHRQoL scores for the Frankel II than for the twin block at all evaluation points [21]. This finding may relate to variations in how outcomes were measured and to the unique tissue-borne design of the Frankel II. Although it appears bulky, much of its structure lies within the buccal vestibule, which reduces interference with speech and may improve comfort, potentially explaining the more favorable patient-reported outcomes [21].

Swallowing and speech difficulties were also reported, but these were usually minor and limited to the early stages of treatment. Both Class II and Class III appliances (activator, twin-block, aesthetic twin-block, trainer, and RMR) were associated with mild, early-stage impairment that resolved over time [15-17,24,25]. Notably, the activator caused slightly greater initial swallowing difficulty than the trainer [15], likely due to its larger size and rigid, bulky acrylic design, which requires longer neuromuscular adaptation. However, these differences were no longer evident after three months, suggesting that swallowing disturbances are transient and negligible in the long term.

On the other hand, the twin-block appliance caused greater speech impairment than the aesthetic twin-block from T2 to T4. This difference can be attributed to its design, in which the acrylic base extends further across the palatal rugae and incorporates additional wire components, such as the labial bow and clasps [24]. Although the consistency of trends across studies strengthens the reliability of these conclusions, interpretation should be tempered by certain limitations, including heterogeneity in outcome measures and variability in follow-up duration. Clinically, these findings indicate that patients can be reassured that any discomfort or functional impairment associated with functional appliances is typically mild, short-lived, and unlikely to have a lasting impact on quality of life.

Sources and Impact of Heterogeneity

Interpretation of patient-reported outcomes proved challenging due to the considerable heterogeneity among included studies. Differences in PROMs instruments, ranging from OHIP-14, COHIP-19, CPQ11-14, and customized questionnaires with varying constructs and score ranges, complicated direct comparisons. In addition, variability in participant age and developmental stage across studies likely influenced patients' perceptions and the reporting of outcomes. Variations in appliance design (e.g., bulky acrylic versus tissue-borne appliances) also plausibly contributed to between-study variability and limited the feasibility of meta-analysis.

Practical Clinical Implications

Clinically, pre-treatment counseling is essential to reassure patients that mild pain and transient swallowing difficulties are expected early but usually resolve within three months [15-17]. Appliance designs with minimal intraoral bulk and vestibular obstruction should be preferred when comfort is a priority. Regular monitoring of PROMs during the first three months helps document adaptation and identify patients needing additional support or adjustment.

Limitation

This review is not without limitations. Several included studies exhibited either unclear or high risk of bias, often due to lack of blinding, incomplete follow-up, or insufficient reporting. reducing. Substantial heterogeneity in PROMs instruments (OHI-14, COHIP-19, CPQ11-14, customized questionnaires), follow-up schedules, and patients' ages prevented quantitative pooling and complicated comparisons. Sample sizes were generally small and clustered in a few centers and countries, limiting external validity. Moreover, most included studies originated from a single geographical region, further limiting the generalizability of the findings. Finally, meta-analysis was not feasible, and publication bias cannot be excluded.

Recommendations for Future Research

To strengthen the evidence base for functional appliance therapy, future research should focus on multicenter RCTs employing standardized PROMs with clearly defined primary outcomes and sample sizes adequately powered to detect minimal clinically important differences. Wherever feasible, allocation concealment and blinded outcome assessment should be implemented, and prospective protocol registration will improve reliability and transparency. The development of a core outcome set for patient-reported outcomes in functional appliance research is also essential to harmonize measurements across studies. Additionally, reporting raw scores along with measures of variance is strongly recommended to facilitate accurate synthesis in future meta-analyses, thereby enhancing comparability and the overall robustness of findings.

## Conclusions

Overall, the available evidence suggests that functional appliances used in Class II and Class III malocclusions are generally associated with mild and transient patient-reported effects, with limited impact on OHRQoL. The certainty of the evidence for these outcomes ranged from low to very low. 

Nevertheless, the strength of these conclusions is restricted by methodological limitations, heterogeneity in outcome measures, and limited external validity. Therefore, further well-designed, adequately powered, and standardized clinical trials are needed to establish more definitive recommendations for clinical practice.

## Appendices

Appendix A

**Table 5:** Electronic search strategy

Database	Search Strategy
Cochrane Central Register of Controlled Trials (CENTRAL)	#1 "class II malocclusion" OR " skeletal class II" OR "distal occlusion" OR "mandibular retrognathia" OR " mandibular retrognathism" OR "class III malocclusion " OR " skeletal class III" OR "Mesial occlusion" OR "mandibular prognathism". #2 "growth modification" OR "functional treatment" OR "functional orthopedic" OR "jaw relationship correction “OR "removable functional appliance" OR" fixed functional appliance" OR "Activator" OR "Frankle regulator "OR "Bionater" OR "Twin block" OR "Herbst" OR "modified Herbst" OR "Hotz" OR "trainers " OR " Double plates" OR "Dynamax" OR "Miniblock “OR"Jasper jumper "OR" Forsus nitinol flat spring" OR "Forsus device "OR" Sabbagh spring “OR" Bimler appliance "OR" Bass appliance" OR "Andresen appliance" OR "RMR" OR " revirse Twin-block" OR "tandem" OR "Bimler". #3 "Quality of life" OR " oral health related quality of life" OR "pain" OR " discomfort" OR " functional impairments" OR "Acceptance" OR "self_confidence" OR "Irritation" OR "Limitation" OR "Mastication" OR "Oral ulcers" OR "Patient experiences" OR "Patient Perception" OR "Patient-reported outcome measures" OR "PROMs" OR "Pressure" OR "Restriction" OR "Soreness" OR "Speech" OR "Swelling" OR "Tension" OR "Self-esteem" OR "edma" OR "paresthesia. #4 #1 AND #2 AND #3
Excerpta Medica database (Embase)	#1 "class II malocclusion" OR " skeletal class II" OR "distal occlusion" OR "mandibular retrognathia" OR " mandibular retrognathism" OR "class III malocclusion " OR " skeletal class III" OR Mesial occlusion" OR "mandibular prognathism". #"growth modification" OR "functional treatment" OR "functional orthopedic" OR "jaw relationship correction “OR "removable functional appliance" OR" fixed functional appliance" OR "Activator" OR "Frankle regulator "OR "Bionater" OR "Twin block" OR "Herbst" OR "modified Herbst" OR "Hotz" OR "trainers " OR " Double plates" OR "Dynamax" OR "Miniblock “OR"Jasper jumper "OR" Forsus nitinol flat spring" OR "Forsus device "OR" Sabbagh spring “OR" Bimler appliance "OR" Bass appliance" OR "Andresen appliance" OR "RMR" OR " revirse Twin-block" OR "tandem" OR "Bimler". #3 "Quality of life" OR " oral health related quality of life" OR "pain" OR " discomfort" OR " functional impairments" OR "Acceptance" OR "self_confidence" OR "Irritation" OR "Limitation" OR "Mastication" OR "Oral ulcers" OR "Patient experiences" OR "Patient Perception" OR "Patient-reported outcome measures" OR "PROMs" OR "Pressure" OR "Restriction" OR "Soreness" OR "Speech" OR "Swelling" OR "Tension" OR "Self-esteem" OR "edma" OR "paresthesia #4 #1 AND #2 AND #3
PubMed	#1 "class II malocclusion" OR " skeletal class II" OR "distal occlusion" OR "mandibular retrognathia" OR " mandibular retrognathism" OR "class III malocclusion " OR " skeletal class III" OR "Mesial occlusion" OR "mandibular prognathism". #2 " growth modification" OR "functional treatment" OR "functional orthopedic" OR "jaw relationship correction “OR "removable functional appliance" OR" fixed functional appliance" OR "Activator" OR "Frankle regulator "OR "Bionater" OR "Twin block" OR "Herbst" OR "modified Herbst" OR "Hotz" OR "trainers " OR " Double plates" OR "Dynamax" OR "Miniblock “OR"Jasper jumper "OR" Forsus nitinol flat spring" OR "Forsus device "OR" Sabbagh spring “OR" Bimler appliance "OR" Bass appliance" OR "Andresen appliance" OR "RMR" OR " revirse Twin-block" OR "tandem" OR "Bimler". #3 "Quality of life" OR " oral health related quality of life" OR "pain" OR " discomfort" OR " functional impairments" OR "Acceptance" OR "self_confidence" OR "Irritation" OR "Limitation" OR "Mastication" OR "Oral ulcers" OR "Patient experiences" OR "Patient Perception" OR "Patient-reported outcome measures" OR "PROMs" OR "Pressure" OR "Restriction" OR "Soreness" OR "Speech" OR "Swelling" OR "Tension" OR "Self-esteem" OR "edma" OR "paresthesia #4 #1 AND #2 AND #3
Scopus	#1TITLE-ABS-KEY (""class II malocclusion " OR " skeletal class II" OR "distal occlusion" OR "mandibular retrognathia" OR " mandibular retrognathism" OR "class III malocclusion " OR " skeletal class III" OR "Mesial occlusion" OR "mandibular prognathism"). #2TITLE-ABS-KEY (""growth modification" OR "functional treatment" OR "functional orthopedic" OR "jaw relationship correction “OR "removable functional appliance" OR" fixed functional appliance" OR "Activator" OR "Frankle regulator "OR "Bionater" OR "Twin block" OR "Herbst" OR "modified Herbst" OR "Hotz" OR "trainers " OR " Double plates" OR "Dynamax" OR "Miniblock “OR"Jasper jumper "OR" Forsus nitinol flat spring" OR "Forsus device "OR" Sabbagh spring “OR" Bimler appliance "OR" Bass appliance" OR "Andresen appliance" OR "RMR" OR " revirse Twin-block" OR "tandem" OR "Bimler"). #3TITLE-ABS-KEY ("Quality of life" OR " oral health related quality of life" OR "pain" OR " discomfort" OR " functional impairments" OR "Acceptance" OR "self_confidence" OR "Irritation" OR "Limitation" OR "Mastication" OR "Oral ulcers" OR "Patient experiences" OR "Patient Perception" OR "Patient-reported outcome measures" OR "PROMs" OR "Pressure" OR "Restriction" OR "Soreness" OR "Speech" OR "Swelling" OR "Tension" OR "Self-esteem" OR "edma" OR "paresthesia). #5 #1 AND #2 AND #3
Web of Science	#1 TS= ("class II malocclusion" OR " skeletal class II" OR "distal occlusion" OR "mandibular retrognathia" OR " mandibular retrognathism" OR "class III malocclusion " OR " skeletal class III" OR "Mesial occlusion" OR "mandibular prognathism"). #2 TS= ("growth modification" OR "functional treatment" OR "functional orthopedic" OR "jaw relationship correction “OR "removable functional appliance" OR" fixed functional appliance" OR "Activator" OR "Frankle regulator "OR "Bionater" OR "Twin block" OR "Herbst" OR "modified Herbst" OR "Hotz" OR "trainers " OR " Double plates" OR "Dynamax" OR "Miniblock “OR"Jasper jumper "OR" Forsus nitinol flat spring" OR "Forsus device "OR" Sabbagh spring “OR" Bimler appliance "OR" Bass appliance" OR "Andresen appliance" OR "RMR" OR " revirse Twin-block" OR "tandem" OR "Bimler". #3 TS= ("Quality of life" OR " oral health related quality of life" OR "pain" OR " discomfort" OR " functional impairments" OR "Acceptance" OR "self_confidence" OR "Irritation" OR "Limitation" OR "Mastication" OR "Oral ulcers" OR "Patient experiences" OR "Patient Perception" OR "Patient-reported outcome measures" OR "PROMs" OR "Pressure" OR "Restriction" OR "Soreness" OR "Speech" OR "Swelling" OR "Tension" OR "Self-esteem" OR "edma" OR "paresthesia"). #5 #1 AND #2 AND #3
Google Scholar	#1")"class II malocclusion" OR " skeletal class II" OR "distal occlusion" OR "mandibular retrognathia" OR " mandibular retrognathism" OR "class III malocclusion " OR " skeletal class III" OR "Miseal occlusion" OR "mandibular prognathism".) AND " )growth modification" OR "functional treatment" OR "functional orthopedic" OR "jaw relationship correction “OR "removable functional appliance" OR" fixed functional appliance" OR "Activator" OR "Frankle regulator "OR "Bionater" OR "Twin block" OR "Herbst" OR "modified Herbst" OR "Hotz" OR "trainers " OR " Double plates" OR "Dynamax" OR "Miniblock “OR"Jasper jumper "OR" Forsus nitinol flat spring" OR "Forsus device "OR" Sabbagh spring “OR" Bimler appliance "OR" Bass appliance" OR "Andresen appliance" OR "RMR" OR " revirse Twin-block" OR "tandem" OR "Bimler") AND ("Quality of life" OR " oral health related quality of life" OR "pain" OR " discomfort" OR " functional impairments" OR "Acceptance" OR "self_confidence" OR "Irritation" OR "Limitation" OR "Mastication" OR "Oral ulcers" OR "Patient experiences" OR "Patient Perception" OR "Patient-reported outcome measures" OR "PROMs" OR "Pressure" OR "Restriction" OR "Soreness" OR "Speech" OR "Swelling" OR "Tension" OR "Self-esteem" OR "edma" OR "paresthesia).
World Health Organization (WHO) International Clinical Trials Registry Platform (ICTRP) Search Portal	(Functional orthodontic treatment or functional orthopedic treatment or mandibular advancement or functional appliance or mandibular protrusion appliance) AND (quality of life).
ClinicalTrials.gov	(Functional orthodontic treatment or functional orthopedic treatment or mandibular advancement or functional appliance or mandibular protrusion appliance) AND (quality of life).

Appendix B

**Table 6:** Details of the risk of bias assessment of randomized controlled trials

Study	Bias arising from the randomization process	Bias due to deviations from intended interventions		Bias due to missing outcome data	Bias in measurement of the outcome	Bias in selection of the reported result	Overall bias
		Effect of assignment to intervention	Effect of adhering to intervention				
	D1	D2		D3	D4	D5	
Idris et al., 2012 [15]	Some concerns: The method used for randomization was not reported. "A sample of 60 skeletal Class II division 1 subjects was recruited and distributed randomly and equally into two groups".	Some concerns: Blinding cannot be performed. There is ‘No information’ on whether any deviations arose because of the trial context.	Some concerns: Blinding cannot be performed. And ‘No information on whether the important non-protocol interventions were balanced across intervention groups.	Low risk: A total of six patients were lost to follow-up (2 patients in the Activator group and 4 in Trainer TK4 group) Nearly all the outcome data is available 90%.	Some concerns: No details of blinding of outcome assessors were reported. The method of measuring the outcome is appropriate.	Low risk: No information about the registration protocol was mentioned. However, the reported outcomes in the results section seemed to correspond with the predefined outcomes stated in the method section.	High risk
Campbell et al., 2020 [23]	Low risk: The method used for randomization was reported. "Randomization was carried out using a computer generated random number sequence in blocks of 10 with an allocation ratio of 1:1".	Some concerns: Blinding cannot be performed. There is ‘No information’ on whether any deviations arose because of the trial context.	Some concerns: Blinding cannot be performed. And ‘No information’ on whether the important non-protocol interventions were balanced across intervention groups.	High risk: A total of eighteen patients were lost to follow-up (10 patients in FR2 group and 8 in MTW group)	Low risk: blinding of outcome assessors were reported. The method of measuring the outcome is appropriate.	Low risk: No information about the registration protocol was mentioned. However, the reported outcomes in the result section seemed to be corresponding with the pre-defined outcomes aforesaid in the method section.	High risk
Pacha et al., 2023 [22]	Low risk: The method used for randomization was reported. "Randomization was accomplished using electronic software with allocation concealed using sequentially numbered, opaque, and sealed envelopes".	Some concerns: Blinding cannot be performed. There is ‘No information’ on whether any deviations arose because of the trial context.	Some concerns: Blinding cannot be performed. And ‘No information on whether the important non-protocol interventions were balanced across intervention groups.	Low risk: No dropouts were reported.	Low risk: blinding of outcome assessors were reported. The method of measuring the outcome is appropriate.	Low risk: No information about the registration protocol was mentioned. However, the reported outcomes in the result section seemed to be corresponding with the pre-defined outcomes aforesaid in the method section.	Some concerns
Alsilq et al., 2025 [24]	Low risk A computer-generated randomization list was used to randomly divide the patients into two equal groups via Minitab® Version 19.1 (Minitab Inc., Pennsylvania, USA), which was created by one of the academic staff (not involved in this research) at the Department of Orthodontics.	Some concerns: Blinding cannot be performed. There is ‘No information’ on whether any deviations arose because of the trial context.	Some concerns: Blinding cannot be performed. And ‘No information on whether the important non-protocol interventions were balanced across intervention groups	Low risk: No dropped out.	Low risk: blinding of the assessment was performed The method of measuring the outcome is appropriate	Low risk The trial was prospectively registered in an international clinical trial registry. The presence of a preregistered protocol reduces the likelihood of selective outcome reporting, and no discrepancies were identified between the reported outcomes and the registered protocol.	Some concerns
Saleh et al., 2013 [17]	Low risk: The method used for randomization was reported “ The randomization procedure of these 72 skeletal Class III patients was done manually by simply asking each participant to pick up a concealed opaque envelope form a black plastic box. …..”	Some concerns: Blinding cannot be performed. There is ‘No information’ on whether any deviations arose because of the trial context.	Some concerns: Blinding cannot be performed. And ‘No information on whether the important non-protocol interventions were balanced across intervention groups.	Low risk: A total of 5 patients were lost to follow-up (3 patients in the RMR group and 2 in control group) Nearly all the outcome data is available 90%.	Some concerns: No details of blinding of outcome assessors were reported. The method of measuring the outcome is appropriate.	Low risk: No information about the registration protocol was mentioned. However, the reported outcomes in the result section seemed to be corresponding with the pre-defined outcomes aforesaid in the method section.	Some concerns
Majanni et al., 2020 [16]	Low risk: The method used for randomization was reported. "56 patients were randomly selected and assigned to the two groups in a 1:1 allocation ratio by creating a randomisation list using Minitab® V16".	Some concerns: Blinding cannot be performed. There is ‘No information’ on whether any deviations arose because of the trial context.	Some concerns: Blinding cannot be performed. And ‘No information on whether the important non-protocol interventions were balanced across intervention groups.	Low risk: A total of 5 patients were lost to follow-up (3 patients in RMR group and 2 in BAIMT group) Nearly all the outcome data is available 90%.	Some concerns: No details of blinding of outcome assessors were reported. The method of measuring the outcome is appropriate.	Low risk: No information about the registration protocol was mentioned. However, the reported outcomes in the result section seemed to be corresponding with the pre-defined outcomes aforesaid in the method section.	Some concerns
Zakaria et al., 2025 [25]	Low risk: The method used for randomization was reported. "The allocation sequence was concealed from the main researche by utilizing sequentially numbered, nontransparent, and sealed envelopes. However, these envelopes were opened prior to the start of orthodontic treatment.".	Some concerns: Blinding cannot be performed. There is ‘No information’ on whether any deviations arose because of the trial context.	Some concerns: Blinding cannot be performed. And ‘No information on whether the important non-protocol interventions were balanced across intervention groups.	Low risk: No dropped out.	Low risk: blinding of the assessment was performed	Low risk The trial was prospectively registered in an international clinical trial registry. The presence of a preregistered protocol reduces the likelihood of selective outcome reporting, and no discrepancies were identified between the reported outcomes and the registered protocol.	Some concerns

Appendix C

**Table 7:** Details of the risk of bias assessment of the cohort study

Study	Risk of bias due to confounding	Risk of bias in selection of participants into the study	Risk of bias in classification of interventions	Risk of bias due to deviations from intended interventions	Risk of bias due to missing data	Risk of bias in measurement of outcomes	Risk of bias in selection of the reported result	Over-all bias
Pakkhesal et al., 2023 [21]	Low risk: No confounding expected	Low risk: All the participants started at the same time, and the start of the intervention coincided	Low risk: Intervention is well defined	Low risk: There were no deviations from the intended intervention beyond what would be expected in usual practice	Low risk: Data were available for all participants	Moderate risk: The research was blinded	Low risk: The measurements and analysis of selected variables were reported in the study.	Moderate risk of bias
